# Rod Origami (RodOri) Spring Metamaterials for Tunable Vibration Control via Tailored Structural Instabilities

**DOI:** 10.1002/advs.76120

**Published:** 2026-06-15

**Authors:** Jeseung Lee, Sophie Leanza, Ruike Renee Zhao

**Affiliations:** ^1^ Department of Mechanical Engineering Stanford University Stanford California USA

**Keywords:** mechanical metamaterials, reconfigurable structures, rod origami, structural instability, vibration control

## Abstract

Modern engineering systems increasingly operate under varying vibrational environments, necessitating structural components capable of adapting their dynamic responses on demand. While springs have long served as the core of vibration control systems, their fixed stiffness fundamentally limits adaptability. Here, we introduce reconfigurable springs based on the structural instability of rod origami (RodOri), constructed from pre‐stressed, naturally curved elastic rods. By tailoring the natural curvature and cross‐sectional aspect ratio of the constituent rods, both the onset of snap‐through buckling and the post‐buckling response of individual RodOri springs can be systematically programmed. Leveraging this geometric programmability, we establish a system‐level design principle in which multiple RodOri springs of identical length but distinct buckling behaviors are assembled into a multistable metamaterial. Differences in snapping displacements enable stepwise structural reconfiguration via sequential snap‐through transitions, while variations in post‐snapping stiffness govern the mechanical response of each configuration. This hierarchical tunability enables both broad and fine control of resonance frequencies and dynamic responses across stable states. Numerical simulations and experiments demonstrate on‐demand modulation of vibration amplification, isolation, and impact mitigation within a single metamaterial, establishing RodOri springs as reconfigurable, programmable building blocks for adaptive structural and wave dynamics.

## Graphical abstract

Rod Origami (RodOri) springs harness snap‐through instabilities of pre‐stressed curved rods with geometrically programmable buckling behavior. Integrating RodOri springs with distinct geometries yields multistable metamaterials that undergo stepwise reconfiguration via sequential snapping, achieving wide‐range tunability of stiffness and resonance frequency, and enabling precise, on‐demand control of vibration isolation, amplification, and impact mitigation.

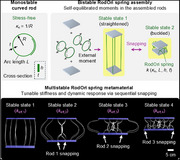

## 1 Introduction

In modern engineering systems, including spacecraft [1, 2], civil infrastructure [3, 4], and industrial robots [5, 6], vibrational environments are not fixed but inherently change with operating conditions, demanding adaptive vibration control. In aerospace engineering, for example, spacecraft structures experience severe vibrations with varying frequencies during launch [7], in contrast to the micro‐vibration‐sensitive conditions encountered during on‐orbit operation [8]. To protect sensitive payloads under these contrasting environments, they employ lock‐release mechanisms that remain highly stiff during launch to suppress excessive deformation yet switch to low‐stiffness, isolation‐optimized configurations once in orbit [9]. Beyond aerospace applications, resonance frequency tuning is also critical in precision mechanical systems such as rotating turbomachinery, where variations in operational speed sweep excitation frequencies across structural resonances, leading to excessive vibration unless the resonance characteristics can be dynamically adjusted [10]. In other systems aimed at energy harvesting or enhanced sensing, vibrations are harnessed as functional resources under varying environmental conditions [11, 12]. These considerations motivate the development of structural elements whose vibration responses can be easily, widely, and reversibly tuned to accommodate dynamically changing operational conditions.

Springs are the most fundamental components of vibration control systems, as they store and release elastic energy under dynamic loading [13]. By properly designing stiffness and mass distribution, springs can achieve passive vibration isolation from external excitations [14, 15], while coupling with damping elements enables efficient energy dissipation [16, 17]. However, conventional springs possess fixed stiffness and therefore provide only a single dynamic response, limiting adaptability under changing operating conditions. Recent advances in architected structures, also known as mechanical metamaterials [18, 19, 20], have enabled tunable stiffness and dynamic responses by exploiting mechanically reconfigurable structures [21, 22, 23, 24, 25] and stimuli‐responsive materials [26, 27, 28, 29]. Nevertheless, most existing systems still rely on complex actuation mechanisms or intricate fabrication processes [30, 31], operate effectively only within narrow tuning ranges [32], or lack reliable in situ reconfigurability [33, 34, 35]. These limitations hinder their practical implementation in adaptive vibration control systems that demand both structural simplicity and robust, wide‐range tunability.

**FIGURE 1:**
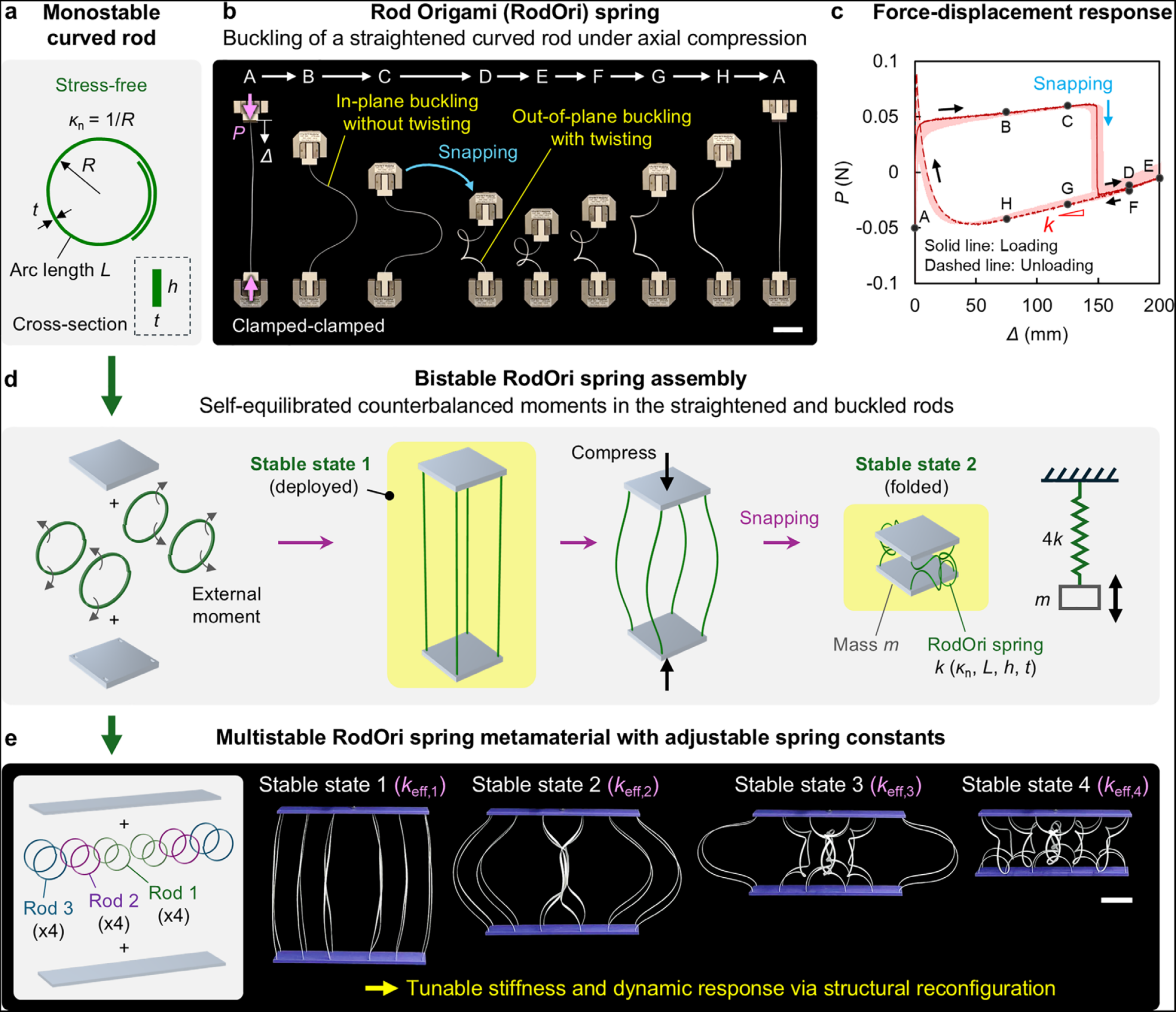
Reconfigurable rod origami (RodOri) springs and metamaterials for tunable stiffness and dynamic responses. (a) Monostable naturally curved elastic rod in its stress‐free state. The geometry is characterized by natural curvature (*κ*_n_ = 1/*R*, where *R* is the stress‐free radius of curvature), arc length (*L*), and cross‐sectional dimensions (height *h* and thickness *t*). (b) Buckling behavior of a straightened RodOri spring (*Lκ*_n_/2*π* = 1.32, *h*/*t* = 4, *L* = 251.3 mm, *t* = 0.6 mm) under axial compression with clamped‐clamped boundary conditions. The representative force‐displacement response is presented with the shaded band indicating the minimum‐maximum range from three repeated measurements. *P*: compressive force, *Δ*: compressive displacement. Scale bar: 5 cm. (c) Corresponding force‐displacement response of the RodOri spring. The slope of the post‐snapping regime defines the effective spring constant *k*. (d) Bistable RodOri spring assembly formed by straightening two pairs of curved rods and connecting them between two rigid frames (mass *m*). The assembly is stable in both its deployed (straightened) and folded (buckled) configurations. (e) Multistable RodOri spring metamaterial with adjustable spring constants. The metamaterial composed of three different types of RodOri springs exhibits four stable configurations, enabling tunable stiffness, vibration control, and impact mitigation. Scale bar: 5 cm.

Here, we introduce multistable rod origami (RodOri) spring metamaterials, whose easily programmable buckled geometries and material properties enable both broad and fine tuning of stiffness and resonance frequency across distinct stable states, providing a reconfigurable platform for adaptive vibration control under dynamically varying conditions. The term “origami” is used to emphasize that the proposed system employs a rod‐based origami‐inspired design principle [36, 37], in which geometry‐driven transformations among deployed and folded configurations are harnessed to achieve tunable mechanical functionality. The core of the RodOri spring is a naturally curved elastic rod (Figure 1a). When the curved rod is straightened and clamped at both ends, it exhibits structural instability under sufficient axial compression [38, 39]. During this process, the rod first undergoes in‐plane buckling and subsequently experiences a snap‐through transition to an out‐of‐plane buckled spring configuration (Figure 1b and Video S1), with the corresponding force‐displacement curve exhibiting a force drop at the snapping point (Figure 1c). After this snap‐through transition, the rod enters a post‐snapping regime, whose slope defines the effective spring constant *k* of the RodOri spring. The rod's geometric parameters, including natural curvature (*κ*_n_ = 1/*R*, where *R* is the stress‐free radius of curvature), arc length (*L*), and cross‐sectional dimensions (height *h* and thickness *t*), govern both the onset of snap‐through buckling and the post‐buckling mechanical response. Consequently, the effective spring constant *k* can be systematically programmed through these geometric parameters.

To exploit the spring constant shift enabled by instability‐driven structural transitions between straightened and buckled configurations, a bistable RodOri spring assembly (Figure 1d) is created by connecting the ends of curved rods with rigid frames. Specifically, two pairs of curved rods are straightened by applying external bending moments in opposite directions and then connected between two rigid frames. The resulting assembly becomes self‐equilibrated due to the counterbalanced internal bending moments of the straightened rods, yielding a stable deployed (straightened) configuration. Under axial compression, the assembly undergoes snap‐through buckling, forming a folded (buckled) configuration that constitutes a second stable state. These deployed and folded stable configurations can be reversibly accessed under axial loading (Video S2).

Furthermore, we exploit the geometric programmability of the buckling behavior of individual RodOri springs, including both the onset of snap‐through buckling and the post‐buckling response, as a system‐level design principle for multistability and tunable functionality. By assembling RodOri springs with the same length but different natural curvatures and/or cross‐sectional aspect ratios, we realize multistable RodOri spring metamaterials that can switch among multiple distinct structural configurations with adjustable spring constants, enabling wide‐range tunability of both static and dynamic mechanical properties. For example, a metamaterial composed of three different types of RodOri springs exhibits four stable states (Figure 1e). Structural transitions among these states produce large, reversible stiffness changes, spanning more than two orders of magnitude, with further fine‐tuning enabled by sequential snapping of the constituent rods. Forced‐vibration and drop‐impact experiments demonstrate that the metamaterial allows on‐demand modulation of resonance frequency, vibration amplification or isolation, and impact mitigation, highlighting that the dynamic response of RodOri spring metamaterials can be reliably reprogrammed by harnessing instability‐driven structural reconfiguration. While previous studies have shown that tunable vibration control can be achieved by combining bistable or multistable units [40, 41, 42, 43], the existing systems often rely on relatively complex structural elements, such as elaborately designed beams, shells, or predefined crease patterns. In contrast, the proposed RodOri spring is built from an extremely simple building block: a naturally curved rod. Moreover, by exploiting the rod's distinctive, geometry‐programmable snap‐through instability, RodOri spring metamaterials achieve multistability and highly tunable mechanical functionality. Therefore, this work establishes RodOri springs as structurally simple yet powerful elements for adaptive mechanical systems, providing promising solutions for advanced vibration control and related dynamic applications.

## 2 Results

### 2.1 Static and Dynamic Mechanical Characterization of Bistable RodOri Spring Assemblies

**FIGURE 2:**
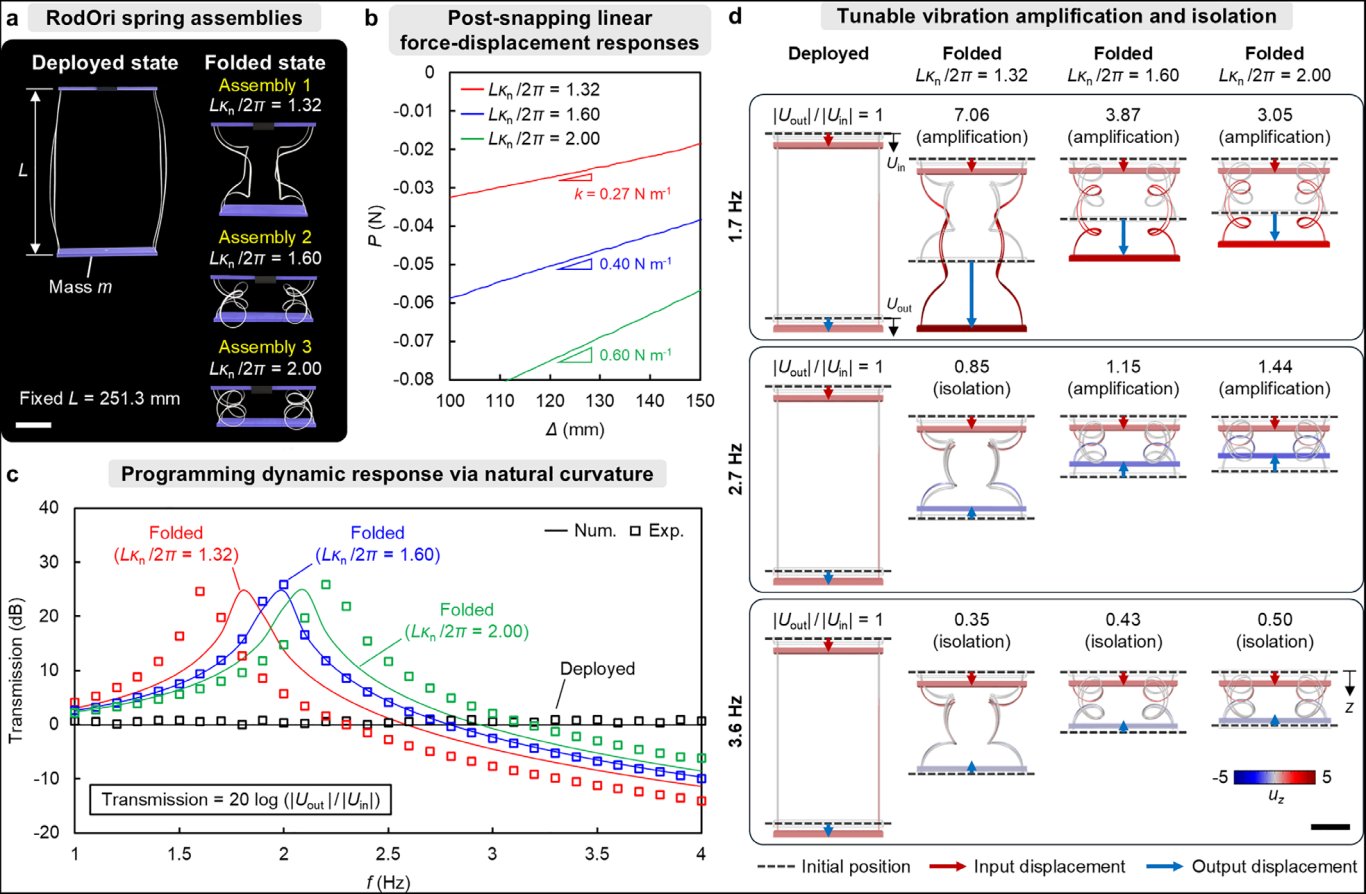
Static and dynamic mechanical characterization of bistable RodOri spring assemblies. (a) RodOri spring assemblies in the deployed (left) and folded (right) stable states. For the folded state, three assemblies with different dimensionless natural curvatures (*Lκ*_n_/2*π* = 1.32 (top), 1.60 (middle), and 2.00 (bottom)) are shown, while keeping *L* = 251.3 mm, *h*/*t* = 4, and *t* = 0.6 mm constant. Scale bar: 5 cm. (b) Post‐snapping linear force‐displacement responses of individual RodOri springs with *Lκ*_n_/2*π* = 1.32 (red), 1.60 (blue), and 2.00 (green) under axial compression with clamped‐clamped boundary conditions. The curves represent the average of three repeated measurements. The slope of each curve defines the effective spring constant *k*. (c) Transmission spectra of the RodOri spring assemblies in the deployed (black) and folded (red, blue, green) states. Vibration transmission is defined as 20 log(|*U*_out_|/|*U*_in_|), where *U*_in_ and *U*_out_ are the input and output displacement amplitudes measured at the top and bottom frames, respectively. The experimental measurements (square markers) are compared with the numerical predictions (solid lines). (d) Numerically simulated displacement fields of the RodOri spring assemblies in the deployed and folded states at 1.7 Hz (top), 2.7 Hz (middle), and 3.6 Hz (bottom). The colormap represents the *z*‐displacement normalized by the input displacement amplitude. Scale bar: 5 cm.

We begin by characterizing the static and dynamic mechanical responses of bistable 4‐rod RodOri spring assemblies, which serve as the fundamental programmable unit of the multistable RodOri spring metamaterial for tunable vibration control. Figure 2a shows the assemblies in the deployed (straightened) and folded (buckled) stable states. This bistability forms the mechanical foundation for shape reconfigurability and configuration‐dependent tunable functionality in the RodOri spring metamaterial.

Because the RodOri spring is composed of a naturally curved elastic rod, the natural curvature in the stress‐free state serves as a key geometric design parameter governing the system's mechanical behavior. Figure 2a (right) shows three sets of assemblies with dimensionless natural curvatures *Lκ*_n_/2*π* = 1.32, 1.60, and 2.00, while keeping other geometric parameters constant (rod length *L* = 251.3 mm, cross‐sectional aspect ratio *h*/*t* = 4, and thickness *t* = 0.6 mm). All rods used in this work are 3D printed with polylactic acid (PLA). Detailed rod fabrication and assembly procedures are provided in Note S1.

Figure 2b presents the post‐snapping linear force‐displacement responses of individual RodOri springs with different natural curvatures, demonstrating that the effective spring constant *k*, characterized by the slope of the force‐displacement curve, increases with increasing natural curvature (see the “Experimental Section”, Note S2, and Figure S1 for experiment details). Quantitatively, increasing *Lκ*_n_/2*π* from 1.32 to 1.60 and 2.00 raises *k* from 0.27 to 0.40 and 0.60 N m^−1^, respectively, demonstrating that natural curvature serves as a powerful geometric parameter for stiffness programming.

We next examine the dynamic response of the RodOri spring assembly to investigate its vibration behavior. In the vibration experiment, the top frame is harmonically excited by a modal shaker while the bottom frame (mass *m* = 10 g) remains free (see the “Experimental Section” for details). Vibration transmission is evaluated as 20 log(|*U*_out_|/|*U*_in_|) as a function of frequency *f*, where *U*_in_ and *U*_out_ are the displacement amplitudes at the top (input) and bottom (output) frames, respectively. The transmission spectra are measured within the frequency range of 1–4 Hz, covering the resonance frequencies of the folded states, with an input displacement amplitude of 1 mm. As shown in Figure 2c, in the deployed state, the straightened rods make the assembly vibrate as a rigid body, resulting in nearly frequency‐independent vibration transmission with |*U*_out_|/|*U*_in_| ≈ 1. In contrast, once the assembly transitions to the folded state, the buckled rods behave as compliant springs. Consequently, the transmission curve shows the characteristic frequency‐dependent behavior of a mass‐spring system, with amplification near resonance and isolation at higher frequencies. Importantly, the resonance frequency increases systematically with *Lκ*_n_/2*π*, consistent with the stiffness variation extracted from the static measurements in Figure 2b. This trend directly follows mass‐spring dynamics, in which a larger stiffness yields a higher resonance frequency. The experimental results (square markers) show good agreement with numerical simulations (solid lines; see Note S3 for simulation details).

Figure 2d further visualizes the simulated spatial deformation fields for the deployed and folded states at 1.7 Hz (top), 2.7 Hz (middle), and 3.6 Hz (bottom), where a larger input amplitude (*U*_in_ = 15 mm) is used to enhance visualization of the vibrational response. In the deployed state, the entire structure moves nearly uniformly at all three frequencies, indicating rigid‐body vibration. In contrast, in the folded state, the system exhibits resonance‐governed vibration dynamics. Notably, the levels of vibration amplification and isolation in the folded state can be programmed through the natural curvature of the constituent rods. For example, at 1.7 Hz, the system exhibits vibration amplification with transmission ratios of 7.06, 3.87, and 3.05 for *Lκ*_n_/2*π* = 1.32, 1.60, and 2.00, respectively, whereas at 3.6 Hz, it exhibits vibration isolation with ratios of 0.35, 0.43, and 0.50. Moreover, at an intermediate frequency of 2.7 Hz, the system transitions between amplification and isolation behavior with ratios of 0.85 (isolation), 1.15 (amplification), and 1.44 (amplification). Overall, the RodOri spring functions as a reconfigurable structural element in which vibration behavior is tuned via snap‐through transitions and programmed via natural curvature.

### 2.2 Programming Buckling Behavior of RodOri Springs via Rod Geometric Parameters

The snap‐through buckling behavior of a RodOri spring can be characterized by three key parameters: the snapping displacement (defined as the compressive displacement at which snap‐through instability occurs), the force drop at the snapping displacement, and the effective spring constant (defined as the local slope of the force‐displacement curve). These parameters play distinct roles in the design of RodOri spring metamaterials exhibiting multistability and tunable mechanical functionality, where the metamaterials are constructed by combining multiple RodOri springs with the same rod lengths but different buckling behaviors. Differences in snapping displacements among the constituent rods enable sequential snap‐through transitions, allowing stepwise structural reconfiguration. The force drop at each snap‐through transition affects whether the snapped configuration of the metamaterial is stable. Meanwhile, the effective spring constants of the constituent rods determine the effective stiffness of the metamaterial in each stable state.

To rationally design these metamaterials, it is essential to understand how rod geometric parameters program the snap‐through buckling behavior of an individual RodOri spring. We systematically vary the dimensionless natural curvature *Lκ*_n_/2*π* from 1.32 to 2.00 and the cross‐sectional aspect ratio *h*/*t* from 4 to 8, while keeping the rod length *L* = 251.3 mm and thickness *t* = 0.6 mm constant (see Figure S2 for fabrication details). The rod length is fixed so that, when assembled into the metamaterial, all rods become straight in the deployed configuration. The buckling behavior is characterized by measuring the force‐displacement response of straightened RodOri springs under axial compression with clamped‐clamped boundary conditions during both loading and unloading (see the “Experimental Section” for details).

**FIGURE 3:**
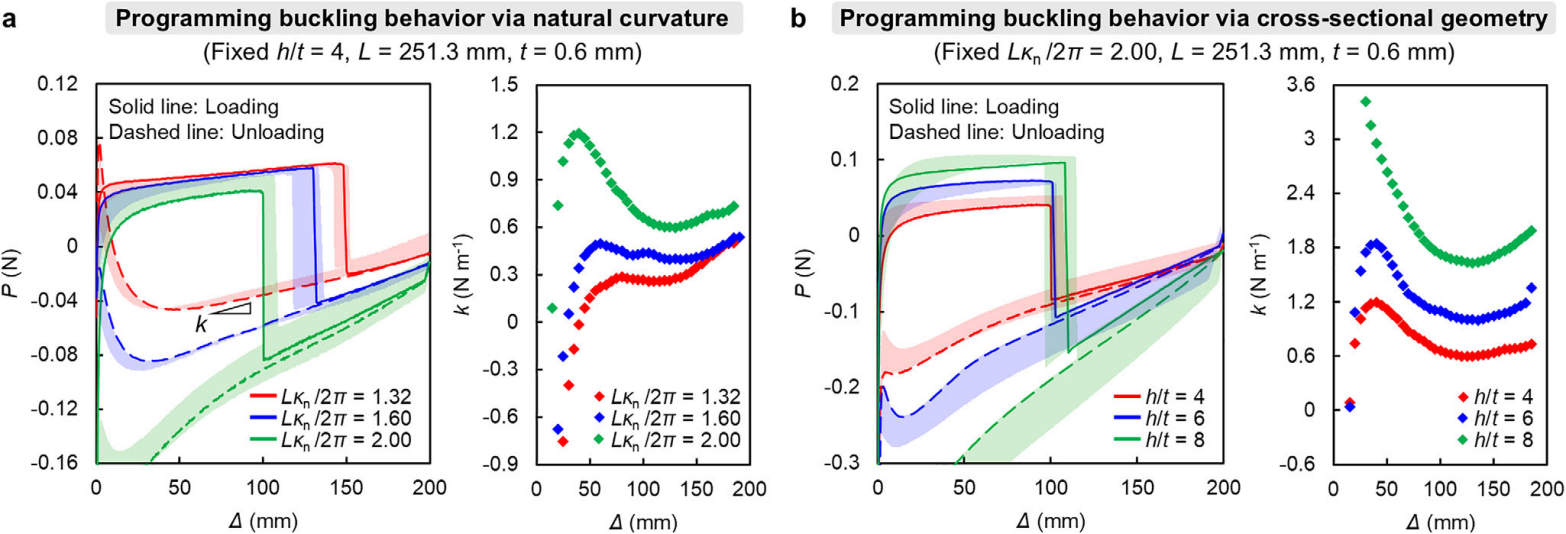
Programming buckling behavior of individual RodOri springs via rod geometric parameters. (a) Force‐displacement curves of RodOri springs with different *Lκ*_n_/2*π* = 1.32 (red), 1.60 (blue), and 2.00 (green), while fixing *h*/*t* = 4. The corresponding effective spring constants, extracted from the local slopes of the unloading curves, are plotted on the right as a function of compressive displacement. (b) Force‐displacement curves of RodOri springs with different *h*/*t* = 4 (red), 6 (blue), and 8 (green), while fixing *Lκ*_n_/2*π* = 2.00. The corresponding effective spring constants, extracted in the same manner, are shown on the right. In both panels, the representative force‐displacement responses are shown with the shaded bands indicating the minimum‐maximum range from three repeated measurements.

Figure 3a compares the buckling behavior of RodOri springs with *Lκ*_n_/2*π* = 1.32, 1.60, and 2.00 at fixed *h*/*t* = 4. Increasing *Lκ*_n_/2*π* not only changes the spring constant (as discussed in the previous section) but, more importantly, systematically shifts the snapping displacement. In particular, rods with larger natural curvature exhibit earlier snapping, demonstrating that the onset of snap‐through instability is programmable through geometric design. This controllable instability can be leveraged to design metamaterials capable of stepwise structural reconfiguration, in which rods with distinct geometries are intentionally combined to snap sequentially (see Figure S3 for details). Notably, as shown in Figure 3a (right), the spring constant *k* depends on the compressive displacement *Δ*: at small *Δ*, *k* exhibits noticeable fluctuations, whereas it progressively stabilizes as compression increases (see Note S2 and Figure S4 for details). This displacement‐dependent spring constant plays a role in tuning the effective stiffness of the metamaterial through structural reconfiguration, as each stable state is formed at a distinct compressive displacement. We further investigate the influence of the cross‐sectional aspect ratio on the buckling behavior of RodOri springs for metamaterial design. Figure 3b compares rods with *h*/*t* = 4, 6, and 8 while fixing *Lκ*_n_/2*π* = 2.00, showing that *h*/*t* also serves as a critical factor in programming the spring constants of individual RodOri springs, with metamaterials constructed from rods with larger *h*/*t* exhibiting increased effective stiffness. Additionally, the magnitude of the force drop during snapping increases with *h*/*t*. In the design of multistable metamaterials, this variation will influence whether the configuration in which the rod has snapped is stable (see Figure S5 for details). Overall, these results demonstrate that the snap‐through buckling behavior of RodOri springs can be systematically programmed through geometric design. Beyond geometry, material selection can provide an additional dimension of programmability in the buckling response (see Note S2 and Figure S6 for details). Here, we note that the force‐displacement curves measured in this study represent the experimentally accessible loading and unloading paths under displacement‐controlled conditions, which are sufficient for characterizing the pre‐buckled and post‐buckled stable states utilized for tunable dynamic functionality in this work. The complete equilibrium path of the RodOri spring is expected to additionally include unstable segments associated with snap‐through behavior, which are not directly captured in the experiments but could be captured using appropriate numerical methods to more fully characterize the nonlinear equilibrium landscape [44].

### 2.3 Multistable RodOri Spring Metamaterials for Tunable Vibration Control

**FIGURE 4:**
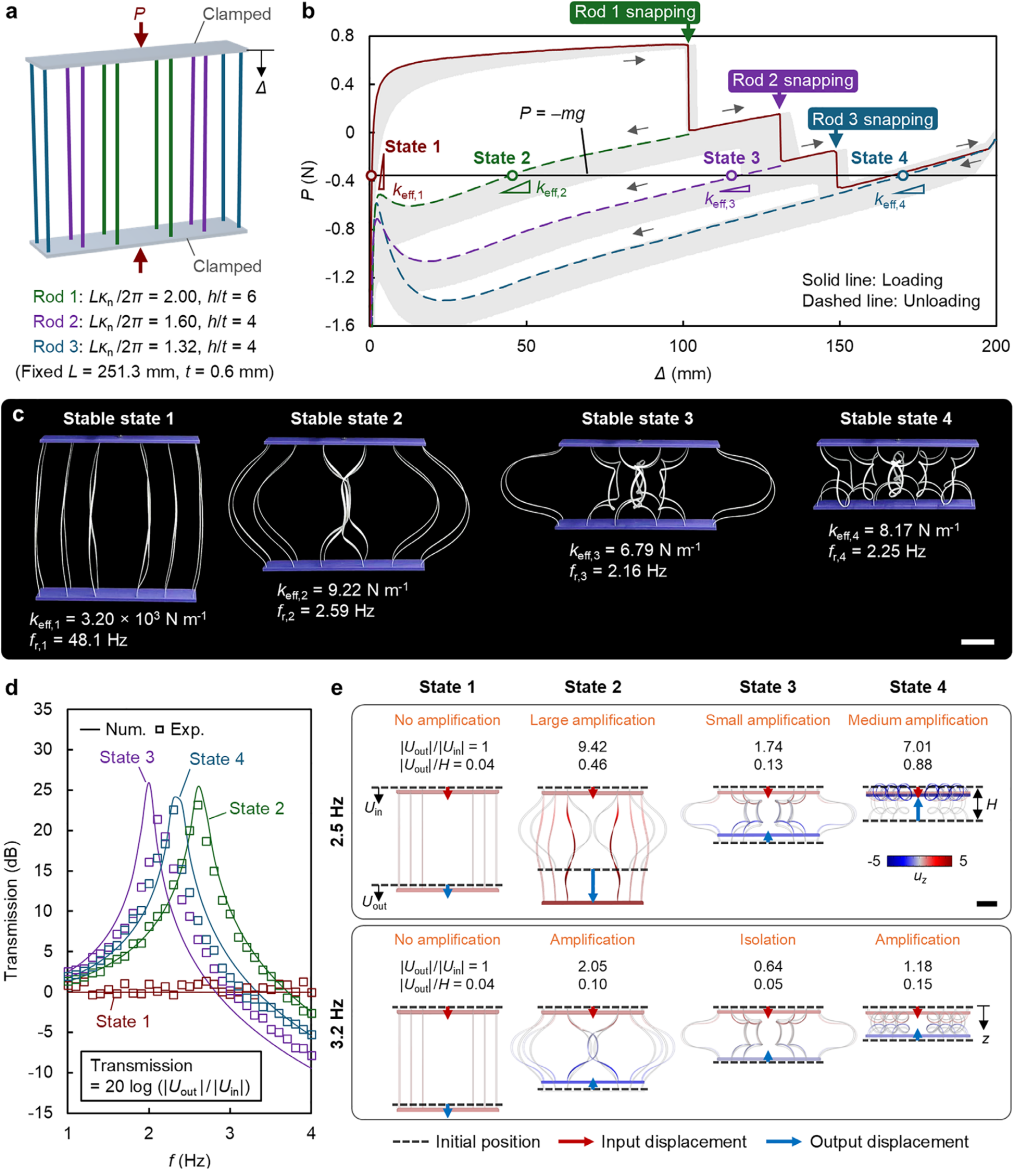
Multistable RodOri spring metamaterials for tunable vibration control. (a) Metamaterial composed of three different types of RodOri springs (rod 1: *Lκ*_n_/2*π* = 2.00, *h*/*t* = 6; rod 2: *Lκ*_n_/2*π* = 1.60, *h*/*t* = 4; rod 3: *Lκ*_n_/2*π* = 1.32, *h*/*t* = 4; fixed *L* = 251.3 mm, *t* = 0.6 mm). (b) Force‐displacement response of the metamaterial under axial compression with clamped‐clamped boundary conditions, obtained by superposing the experimentally measured responses of the constituent RodOri springs. The representative response is presented with the shaded band indicating the minimum‐maximum range from three repeated measurements. One loading curve (red) and three unloading curves (green, purple, and blue), corresponding to sequential snapping of rod types 1, 2, and 3, generate four stable states with distinct effective stiffnesses (*k*_eff,1_, *k*_eff,2_, *k*_eff,3_, *k*_eff,4_). The equilibrium points for vibration experiments (circular markers) are determined by the intersections between the force‐displacement curves and the gravitational load applied by the bottom frame (*P* = –*mg*). (c) Four stable configurations of the metamaterial, each characterized by distinct effective stiffness and corresponding resonance frequency (*f*_r,1_, *f*_r,2_, *f*_r,3_, *f*_r,4_). Scale bar: 5 cm. (d) Vibration transmission spectra for the four stable states. *U*_in_ and *U*_out_ denote the input and output displacement amplitudes measured at the top and bottom frames, respectively. Experimental results (squares) are compared with numerical predictions (solid lines). (e) Tunable vibration control enabled by structural reconfiguration. Numerically simulated displacement fields at 2.5 Hz (top) and 3.2 Hz (bottom) are shown for the four stable states. *H* denotes the structural height of the metamaterial in each state. Colormaps represent the *z*‐displacement normalized by the input displacement amplitude. Scale bar: 5 cm.

By leveraging the geometric programmability of the snapping displacements, force drops, and effective spring constants of individual RodOri springs, multistability and tunable functionality can be embedded in a RodOri spring metamaterial by combining multiple rods of distinct geometries. We construct a metamaterial composed of three different types of rods (rod 1: *Lκ*_n_/2*π* = 2.00, *h*/*t* = 6; rod 2: *Lκ*_n_/2*π* = 1.60, *h*/*t* = 4; rod 3: *Lκ*_n_/2*π* = 1.32, *h*/*t* = 4; fixed *L* = 251.3 mm, *t* = 0.6 mm), as shown in Figure 4a. The rods are connected between two rigid frames (*m* = 35 g), and the fixed rod length ensures that all rods are straight in the deployed configuration. Upon axial compression, based on the single‐rod characterization in Figure 3, rod type 1 snaps first at a compressive displacement of approximately 100 mm, followed by rod type 2 at 130 mm and rod type 3 at 150 mm (see Figure S3 and Video S3 for details). Rod type 1 is intentionally designed with a higher cross‐sectional aspect ratio (*h*/*t* = 6) than rod types 2 and 3 (*h*/*t* = 4) to ensure that all intermediate configurations generated by the three sequential snap‐through transitions remain stable (see Figure S5 for details).

Figure 4b shows the corresponding force‐displacement response of the metamaterial (see the “Experimental Section” and Note S2 for details). The initial loading curve at a near‐zero compressive displacement defines stable state 1, corresponding to the deployed configuration in which all rods remain straight. As compression increases, the loading curve exhibits three distinct force drops, each corresponding to the snapping of rod types 1, 2, and 3. Following each snapping event, unloading is performed to reach stable states 2, 3, and 4, located at compressive displacements of approximately 45, 115, and 170 mm, respectively (indicated by circular markers in Figure 4b). Figure 4c shows the deployed configuration (state 1) together with the folded configurations (states 2–4), in which rod types 1–3 have sequentially undergone snap‐through buckling (see Video S4 for the reconfiguration among the four stable states).

The effective stiffness of each state (*k*_eff,1_, *k*_eff,2_, *k*_eff,3_, *k*_eff,4_) is determined from the local slope of the force‐displacement curve at the equilibrium point. Notably, the deployed state (state 1) is two orders of magnitude stiffer than the folded states (states 2–4), demonstrating a remarkable stiffness tunability ranging from 3.20 × 10^3^ to 6.79 N m^−1^. Furthermore, transitions among the folded states enable additional fine control within the low‐stiffness regime; for example, reconfiguring from states 2 to 3 reduces *k*_eff_ by 26.4%, from 9.22 to 6.79 N m^−1^. This stiffness tunability directly translates into reprogrammable dynamic behavior, as a larger stiffness leads to a higher resonance frequency in a mass‐spring system. Consequently, the four stable states exhibit distinct resonance frequencies (*f*_r,1_, *f*_r,2_, *f*_r,3_, *f*_r,4_). To experimentally validate this tunability, we perform forced‐vibration tests in which the top frame is excited by a modal shaker with the input displacement amplitude *U*_in_ = 1 mm, while the bottom frame remains free to measure the output displacement amplitude *U*_out_ (see the “Experimental Section” and Figure S7 for details). The transmission spectra are characterized over a frequency range of 1–4 Hz, covering the resonance frequencies of the folded states. In the deployed configuration (state 1; red in Figure 4d), the high stiffness yields a resonance frequency of *f*_r,1_ = 48.1 Hz, far above the measured frequency range, resulting in a nearly frequency‐independent transmission curve (|*U*_out_|/|*U*_in_| ≈ 1). In contrast, once the rods buckle into folded configurations (states 2–4; green, purple, and blue in Figure 4d), the substantially reduced stiffness shifts the resonance frequency downward by more than an order of magnitude, producing frequency‐dependent transmission curves. Furthermore, reconfiguration among these folded states enables fine‐tuning of the resonance peak, with *f*_r,2_ = 2.59 Hz, *f*_r,3_ = 2.16 Hz, and *f*_r,4_ = 2.25 Hz, consistent with the stiffness hierarchy identified in Figure 4b.

The demonstrated broad and fine tunability in stiffness and resonance frequency can provide a hierarchical adaptive mechanical functionality for practical applications. For example, in vibration‐based sensing systems [45, 46], adaptive functionality is essential because both the ambient vibration environment and target signal characteristics can vary over time. Here, broad‐tuning enables switching between distinct functional modes, from a high‐stiffness state for rigid load‐bearing to a low‐stiffness state for effective vibration control. However, even after this broad‐tuning, small mismatches between the resonance and excitation frequencies can lead to substantial performance degradation [47]. Fine‐tuning then enables precise alignment of the resonance frequency with the excitation, allowing a single system to maintain optimal resonance‐driven functionality, either to selectively amplify weak target signals or to maximize isolation of unwanted noise, under uncertain and dynamically varying conditions. A quantitative comparison between the proposed RodOri metamaterials and previously reported multistable metamaterials for tunable vibration control [40, 41, 42, 43] is provided in Note S4 and Figure S8, using the normalized frequency‐tuning range and the number of stable states as performance metrics. The comparison shows that our system uniquely combines a broad normalized tuning range and multiple stable states, enabling both broad‐range and fine‐resolution tunability within a structurally simple platform.

The configuration‐dependent resonance frequency directly enables tunable vibration control at targeted excitation frequencies (Video S5). Numerical simulations in Figure 4e illustrate the spatial deformation fields for the four stable states of the metamaterial, with a larger input amplitude (*U*_in_ = 10 mm) to clearly visualize the vibrational response. At 2.5 Hz, the vibration amplification varies markedly across the four states, with |*U*_out_|/|*U*_in_| = 1 (state 1; no amplification), 9.42 (state 2; large amplification), 1.74 (state 3; small amplification), and 7.01 (state 4; medium amplification). Notably, significant amplification is achieved in both states 2 and 4 despite their distinct structural heights (*H* = 206.8 mm and 79.8 mm, respectively), indicating that the system height can be tuned while maintaining comparable dynamic functionality. Moreover, functional switching between vibration amplification and isolation is achieved at 3.2 Hz. Specifically, reconfiguration from states 1 to 2 results in vibration amplification (|*U*_out_|/|*U*_in_| = 2.05), whereas reconfiguration to state 3 leads to vibration isolation (|*U*_out_|/|*U*_in_| = 0.64). Representative input and output displacement time histories at 2.5 and 3.2 Hz are provided in Figure S9, which further support the amplification and isolation characteristics identified from the frequency‐domain spectra.

### 2.4 Multistable RodOri Spring Metamaterials for Tunable Impact Mitigation

**FIGURE 5:**
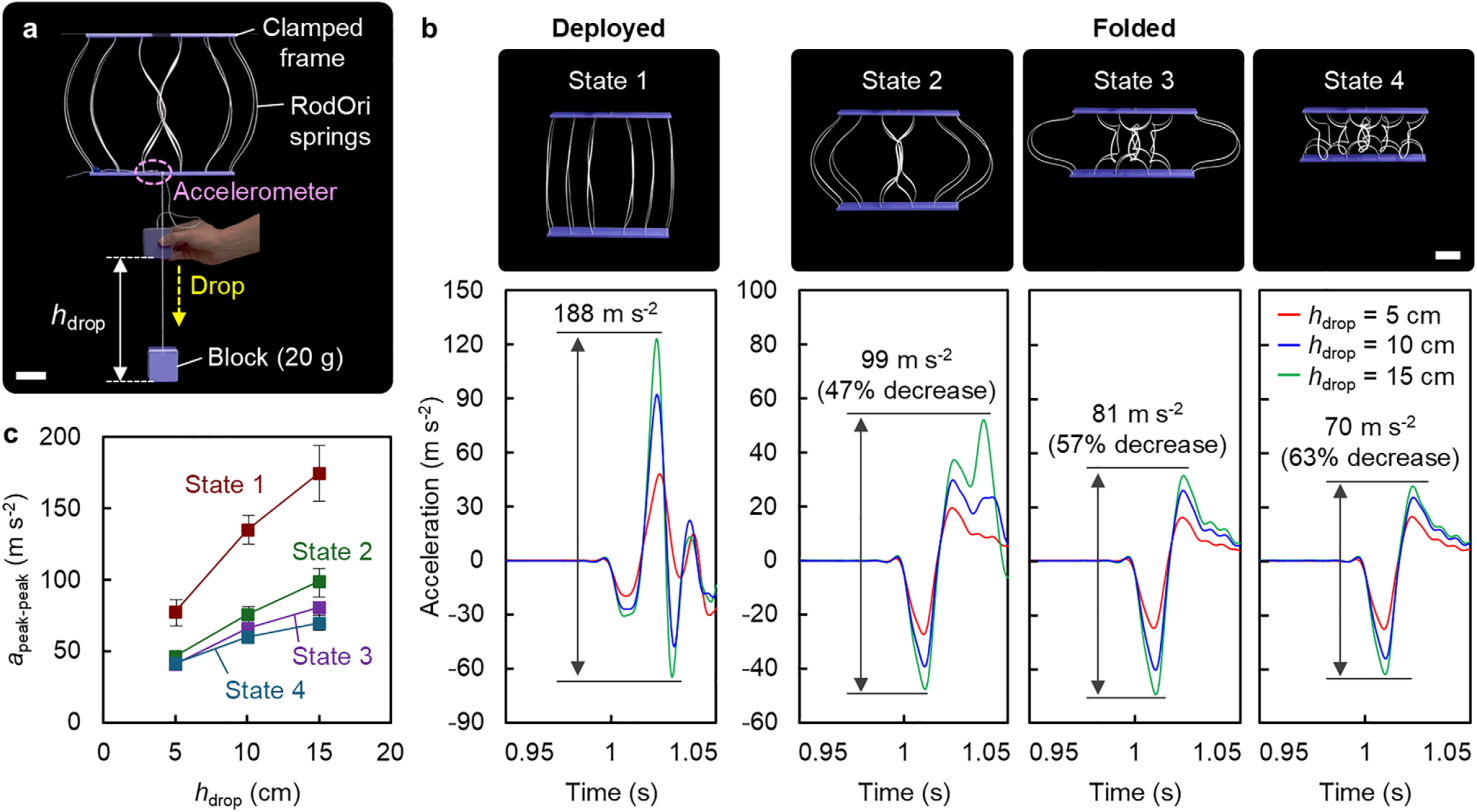
Multistable RodOri spring metamaterials for tunable impact mitigation. (a) Schematic of the drop‐impact experiments. The top frame of the metamaterial (same design as in Figure 4) is rigidly clamped, while the bottom frame is connected to a freely dropping block through a rope. The block is released from a prescribed drop height (*h*_drop_). An accelerometer attached to the bottom frame records the transient acceleration response during impact. Scale bar: 5 cm. (b) Representative acceleration‐time responses during impact for *h*_drop_ = 5 cm (red), 10 cm (blue), and 15 cm (green) across the four stable states. The impact event is temporally aligned at 1 s for direct comparison. The peak‐to‐peak acceleration amplitude (*a*_peak‐peak_) is calculated from the response corresponding to *h*_drop_ = 15 cm. Scale bar: 5 cm. (c) State‐dependent impact‐mitigation performance. The peak‐to‐peak acceleration amplitude during impact is presented as a function of drop height for the four stable states. Error bars denote the standard deviation from three repeated measurements.

Figure 5 demonstrates that the multistable RodOri spring metamaterial also functions as a reconfigurable platform for tunable impact mitigation. To experimentally verify this tunability, we perform controlled drop‐impact tests using a custom‐designed setup (Figure 5a) with the same metamaterial introduced in Figure 4. The top frame is fixed, while the bottom frame is connected to a freely dropping impact block through a rope. The block is released from a designated drop height (*h*_drop_), where the input impact energy is tuned by adjusting *h*_drop_. An accelerometer attached to the bottom frame records the transient acceleration response during impact (see the “Experimental Section” for details). We note that no snap‐through transition occurs under the impact conditions considered here, and thus the metamaterial remains in the same stable state before and after the impact.

Figure 5b compares the representative acceleration‐time responses for the four stable configurations at varied *h*_drop_ = 5, 10, and 15 cm, revealing significant differences in impact‐mitigation capabilities. In the deployed configuration (state 1), the rods remain straightened, limiting large structural deformation. As a result, the metamaterial behaves as a rigid load path, producing a sharp, high‐magnitude acceleration spike. In contrast, once the metamaterial is switched into the folded configurations (states 2–4), the buckled rods undergo large transient deformation during impact. This large deformation increases the duration of the impact event, spreading the impact energy over a longer time and dissipating part of it through internal friction, thereby reducing the transmitted acceleration. Notably, at *h*_drop_ = 15 cm, the peak‐to‐peak acceleration amplitude (*a*_peak‐peak_), defined as the difference between the maximum and minimum acceleration during impact and subsequent rebound, decreases by 63%, from 188 m s^−2^ (state 1) to 70 m s^−2^ (state 4). This metric quantifies the overall shock severity transmitted through the structure.

The tunable impact‐mitigation capability is further explored in Figure 5c. As expected, *a*_peak‐peak_ increases with *h*_drop_ for all configurations due to higher input impact energy. However, the transmitted impact progressively decreases as the metamaterial is reconfigured from state 1 to states 2, 3, and 4, demonstrating stepwise tunable impact‐mitigation performance. Consequently, the RodOri spring metamaterial enables functional switching from a rigid load‐bearing mode (state 1) to an impact‐mitigation mode (states 2–4) with tunable performance. Although not explored in this section, stronger impact conditions capable of triggering snap‐through instability may introduce additional tunability via energy dissipation or release during state transitions, suggesting new possibilities for adaptive impact‐management applications subjected to large transient loads.

## 3 Conclusions

In this work, we have introduced RodOri springs, constructed from pre‐stressed, naturally curved elastic rods, as a new class of reconfigurable structural elements that exploit the geometric programmability of snap‐through buckling. By tailoring the natural curvature and cross‐sectional aspect ratio of the constituent rods, both the onset of snap‐through buckling and the post‐buckling response of individual RodOri springs can be systematically programmed. Leveraging this programmability, we establish a system‐level design principle in which multiple RodOri springs of identical length but distinct buckling behaviors are assembled to realize multistable RodOri spring metamaterials. Differences in snapping displacements enable sequential snap‐through transitions, while variations in post‐snapping stiffness govern the mechanical response of each configuration. As a result, the metamaterial undergoes reversible switching among multiple stable states with markedly distinct properties. This framework enables hierarchical tuning of mechanical behavior, combining broad and fine tuning across discrete stable states. Consequently, a single metamaterial can achieve wide‐range, on‐demand control of vibration amplification, isolation, and impact mitigation.

Beyond the system demonstrated here, increasing the number and diversity of constituent rods will enable access to a larger number of stable configurations and a broader range of tunable mechanical properties. Incorporating materials with different elastic moduli can further expand the design space, offering additional control over both stiffness and dynamic response. Moreover, integrating stimuli‐responsive materials can enable active modulation of buckling behavior [48, 49], transforming the current platform into a more adaptive vibration‐control system with potentially continuous tunability over a wider frequency range. This generalizable framework opens new opportunities for adaptive mechanical systems, including robotics, mechanical logic and computing, morphing structures, and biomedical devices.

## 4 Experimental Section

### 4.1 Rod Fabrication

The naturally curved elastic rods were fabricated via fused‐filament 3D printing (X1 Carbon, Bambu Lab, China) using polylactic acid (PLA Basic, Bambu Lab, China). Each rod was geometrically modeled as a planar spiral to represent the prescribed natural curvature (see Figure S2 for details). Throughout this study, the length and thickness of all rods were fixed at *L* = 251.3 mm and *t* = 0.6 mm, respectively. The dimensionless natural curvature *Lκ*_n_/2*π* was varied from 1.32 to 2.00, while the cross‐sectional aspect ratio *h*/*t* was varied from 4 to 8. Additional fabrication details are provided in Note S1.

### 4.2 Mechanical Testing

Quasi‐static cyclic compression tests were performed using a universal testing machine (Instron 3344, Instron, Inc., USA). The compressive force and displacement were recorded under displacement‐controlled loading and unloading at a rate of 100 mm min^−1^. Each experiment was repeated three times under identical conditions, and the results were presented using representative curves with shaded bands indicating the minimum‐maximum range obtained from repeated measurements. The effective RodOri spring constant was extracted from the averaged local slope of the force‐displacement curve during unloading (see Figure S4 for details). For the RodOri spring metamaterials, the overall force‐displacement response was obtained by superposing the experimentally measured responses of the individual constituent RodOri springs (see Figure S10 for details). Additional experimental details are provided in Note S2.

### 4.3 Forced‐Vibration Testing

Forced‐vibration experiments were performed using a modal shaker (Model JZK‐2, Sinocera Piezotronics, China) driven by a function generator (HP 33120A, Keysight Technologies, USA) and a power amplifier (YE5871A, Sinocera Piezotronics, China). The top frame of the RodOri spring metamaterial was mounted to the shaker (see Figure S7 for details). Harmonic excitation was applied at discrete frequencies ranging from 1 to 4 Hz in steps of 0.1 Hz. Accelerometers (Model 352A21, PCB Piezotronics, USA) were attached to both the top and bottom frames to measure the input (top) and output (bottom) vibrational signals. Data were acquired by a signal conditioner (Model 482C05, PCB Piezotronics, USA) and an oscilloscope (EDUX1002A, Keysight Technologies, USA). The transmission spectrum was computed from the measured acceleration amplitudes.

### 4.4 Impact Testing

Impact‐mitigation experiments were conducted using a custom‐designed drop‐impact setup. The top frame of the RodOri spring metamaterial was clamped, while the bottom frame was connected to a freely dropping block via a rope. Upon impact, transient acceleration responses were measured using an accelerometer (Model 352A21, PCB Piezotronics, USA) mounted on the bottom frame. The signal was recorded with a signal conditioner (Model 482C05, PCB Piezotronics, USA) and an oscilloscope (EDUX1002A, Keysight Technologies, USA). Each test was repeated three times, and the results were reported as mean values with standard deviation error bars (see Figure S11 for details).

### 4.5 Finite Element Simulation

Finite element simulations were performed using COMSOL Multiphysics 6.1 (COMSOL Inc., USA) with the Solid Mechanics module and ABAQUS 2024 (Dassault Systèmes, France). The buckled configurations of the RodOri springs were first obtained using ABAQUS through geometrically nonlinear analysis [39]. Subsequently, the simulated buckled configurations were imported into COMSOL as the geometries for computing the dynamic response of the RodOri spring systems (see Figure S12 for details). The material was modeled as a linear elastic solid with properties corresponding to PLA: Young's modulus of 2.6 GPa, mass density of 1240 kg m^−3^, Poisson's ratio of 0.33, and isotropic loss factor of 0.05 to account for structural damping. Boundary conditions were defined to match the forced‐vibration experimental setup, with harmonic displacement excitation applied at the top frame and the bottom frame allowed to respond dynamically. To account for the natural curvature‐induced pre‐stress in the rods, a bilayer model with opposite thermal expansion coefficients was employed [50]. Frequency‐domain harmonic analysis was conducted to simulate the steady‐state vibration response of the pre‐stressed structure. The transmission spectrum was calculated from the simulated displacement amplitudes of the top (input) and bottom (output) frames. Additional simulation details are provided in Note S3.

## Author Contributions

**Jeseung Lee**: conceptualization, writing – original draft, methodology, investigation, validation, visualization, formal analysis. **Sophie Leanza**: conceptualization, investigation, methodology, visualization, validation, writing – review and editing, formal analysis. **Ruike Renee Zhao**: conceptualization, writing – review and editing, project administration, supervision, funding acquisition, validation.

## Conflicts of Interest

The authors declare no conflicts of interest.

## Supporting information




**Supporting File 1**: advs76120‐sup‐0001‐SuppMat.docx.

**Supporting File 2**: advs76120‐sup‐0002‐MovieS1.mp4.

**Supporting File 3**: advs76120‐sup‐0003‐MovieS2.mp4.

**Supporting File 4**: advs76120‐sup‐0004‐MovieS3.mp4.

**Supporting File 5**: advs76120‐sup‐0005‐MovieS4.mp4.

**Supporting File 6**: advs76120‐sup‐0006‐MovieS5.mp4.

## Data Availability

The data that support the findings of this study are available from the corresponding author upon reasonable request.
