# Supplementary material for: Rod Origami (RodOri) Spring Metamaterials for Tunable Vibration Control via Tailored Structural Instabilities
Source: Adv Sci (Weinh). 2026 Jun 15:e76120. Online ahead of print. doi: 10.1002/advs.76120 (PMC13336391; doi:10.1002/advs.76120)
Supplement: Supplementary file 1 — Supporting File 1: advs76120‐sup‐0001‐SuppMat.docx. [file ADVS-9999-e76120-s001.docx]

Supporting Information

**Rod Origami (RodOri) Spring Metamaterials for Tunable Vibration Control via Tailored Structural Instabilities**

Jeseung Lee, Sophie Leanza, Ruike Renee Zhao*

Department of Mechanical Engineering, Stanford University, Stanford, CA 94305, USA

*Corresponding author. Email: [rrzhao@stanford.edu](mailto:rrzhao@stanford.edu)

**Table of Contents**

Supplementary Notes 1-4

Supplementary Figures S1-S12

Supplementary Videos S1-S5

References

**Supplementary Notes**

**1. Details on rod fabrication and assembly**

This section describes the fabrication and assembly procedures of the RodOri spring metamaterials. All structural components, including the naturally curved elastic rods and rigid rod-connecting frames, were fabricated via 3D printing (X1 Carbon, Bambu Lab, China) using PLA filament. To fabricate rods with a dimensionless natural curvature *Lκ*_n_/2*π* > 1, corresponding to geometries containing more than one loop, each rod was designed as a planar spiral with an approximately constant radius, as shown in **Figure S2a**. Representative 3D-printed rods with various dimensionless natural curvatures (*Lκ*_n_/2*π* = 1.32, 1.60, 2.00) and cross-sectional aspect ratios (*h*/*t* = 4, 6, 8) are shown in **Figure S2b**. For assembly, custom-designed rigid rod-connecting frames are used. Each frame contains rectangular slots designed to securely hold the printed rods in place. To achieve self-equilibrated internal bending moments in the assembled structures, the rods were arranged symmetrically with respect to the two in-plane axes of the frame.

**2. Details on mechanical testing**

This section describes the quasi-static cyclic compression experiments conducted to characterize the mechanical response of the RodOri spring. All tests were conducted using a universal testing machine (Instron 3344, Instron Inc., USA). Prior to each test, the naturally curved rod was mechanically straightened and clamped between two rigid fixtures to impose the clamped-clamped boundary conditions. The compressive force (*P*) and displacement (*Δ*) were recorded throughout each loading-unloading cycle under displacement-controlled settings at a rate of 100 mm min^-1^. The effective RodOri spring constant (*k*) reported in **Figure 3** was determined from the slope of selected unloading segments of the force-displacement curve. Each segment spanned a 20 mm interval of displacement; for example, at *Δ* = 50 mm, the fitting window ranged from 40 to 60 mm. **Figure S4** presents representative local responses of the RodOri spring (*Lκ*_n_/2*π* = 1.32, *h*/*t* = 4, *L* = 251.3 mm, *t* = 0.6 mm) at six compressive displacements (*Δ* = 35, 50, 65, 100, 115, 130 mm). For each displacement, the corresponding unloading segment was fitted using linear least-squares regression, and the slope of the fitted line was defined as the spring constant.

To evaluate repeatability, three RodOri springs with identical geometry (*Lκ*_n_/2*π* = 1.32, *h*/*t* = 4, *L* = 251.3 mm, *t* = 0.6 mm) were each tested over three loading-unloading cycles. **Figure S1a** shows a total of nine force-displacement curves, all consistently exhibiting the characteristic mechanical response associated with snap-through buckling. Minor variations among the curves arise from small geometric imperfections introduced during 3D printing and slight variations in clamping conditions between the rods and fixtures. Importantly, as shown in **Figure S1b**, the slope of the post-snapping unloading segment, corresponding to the spring constant, remains nearly identical across all measurements.

To verify the programmability of the RodOri spring constant through material selection, additional experiments were performed on RodOri springs made of stainless steel and PLA with identical geometric parameters (*Lκ*_n_/2*π* = 2.00, *h*/*t* = 4, *L* = 628 mm, *t* = 0.5 mm). The corresponding Young’s moduli are *E* = 200 GPa for stainless steel and *E* = 2.6 GPa for PLA. For direct comparison, the measured compressive force and displacement were normalized into dimensionless forms as *PL*^2^/*EI* and *Δ*/*L*, where *I* = *ht*^3^/12 is the second moment of area. The normalized responses shown in **Figure S6** indicate that the general mechanical response is similar across different materials, while the onset of snap-through instability and degree of nonlinearity vary between materials.

For the RodOri spring metamaterial, direct measurement of the global force-displacement response was experimentally challenging because sufficient testing space is required to avoid unintended contacts both among neighboring rods and between the rods and the testing machine during loading, which may introduce additional constraints and friction effects into the measured response. In our testing setup, these contacts could not be fully eliminated for the designed RodOri spring metamaterial. Therefore, the metamaterial’s force-displacement curve was estimated by superposing the experimentally measured responses of the individual constituent RodOri springs. To validate this approach, we tested a smaller 3-rod assembly composed of three different rods (rod 1: *Lκ*_n_/2*π* = 2.00, *h*/*t* = 6; rod 2: *Lκ*_n_/2*π* = 1.60, *h*/*t* = 4; rod 3: *Lκ*_n_/2*π* = 1.32, *h*/*t* = 4; fixed *L* = 251.3 mm, *t* = 0.6 mm) that could be measured without contact interference. The directly measured loading and unloading curves of this assembly showed close agreement with the corresponding superposed curves obtained from the individual rod responses (**Figure S10**), supporting the use of the superposition method for the larger RodOri spring metamaterial.

**3. Details on finite element simulations**

This section describes the finite element simulations used to compute the buckling configurations and vibrational responses of the RodOri spring metamaterials. The post-buckled configuration of a single RodOri spring was first obtained using ABAQUS 2024 (Dassault Systèmes, France). In the ABAQUS simulations, the rod was modeled as a straight elastic rod subjected to axial compression under clamped-clamped boundary conditions. To account for the natural curvature-induced pre-stress in the rods, each rod was modeled as a thermally stressed bilayer across its thickness; one layer was assigned a negative coefficient of thermal expansion, while the other layer was assigned an equal positive coefficient (see Ref. [1] for additional details). To simulate the buckling of the rod, one end of the rod was fixed while a uniaxial compressive displacement (rotationally constrained) was imposed on the other end. To trigger instability, a small initial geometric imperfection was introduced prior to compression by twisting one end of the rod by a small amount (see Ref. [2] for additional details).

The buckled geometries obtained from ABAQUS were imported into COMSOL Multiphysics 6.1 (COMSOL Inc., USA) and analyzed using the Solid Mechanics module. **Figure S12** shows good geometric agreement between the numerical and experimental configurations of the RodOri spring metamaterial. The material was modeled as a linear elastic solid with properties corresponding to PLA: Young’s modulus of 2.6 GPa, mass density of 1240 kg m^-3^, Poisson’s ratio of 0.33, and an isotropic structural loss factor of 0.05 to represent damping. The rods were again modeled as thermally stressed bilayers through their thickness. In the COMSOL simulations, a frequency-domain study was carried out using the pre-stressed structure as the linearization point to compute the vibrational response under small harmonic excitation. The prescribed displacement was applied to the top frame, while the remaining boundaries were left free to respond dynamically. The transmission spectrum was calculated from the ratio of displacement amplitudes of the bottom (output) and top (input) frames.

**4. Performance comparison between the proposed and existing multistable metamaterials for tunable vibration control**

This section presents a quantitative performance comparison between the proposed RodOri spring metamaterials and previously reported multistable metamaterials for tunable vibration control (Refs. [40-43] in the main text) using two performance metrics: (1) the normalized frequency-tuning range (Δ*f* / *f*_lowest_, where Δ*f* is the achievable resonance-frequency tuning range and *f*_lowest_ is the lowest accessible resonance frequency), and (2) the number of stable states. Here, a larger Δ*f* / *f*_lowest_ indicates a wider achievable tuning range relative to the lowest accessible resonance frequency. A larger number of stable states indicates finer discrete tunability because it enables more mechanically accessible intermediate configurations. Experimental results reported in the literature are considered in this comparison. As summarized in **Figure S8**, the proposed RodOri spring metamaterial uniquely combines the advantages of a broad normalized tuning range with multiple stable states, enabling both wide-range and fine-resolution tunability within a single structurally simple platform.

**
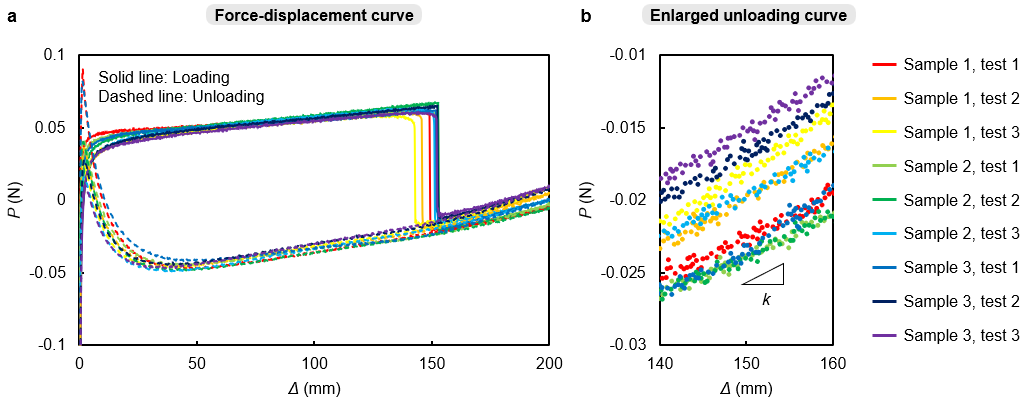
**

**Figure S1.** Repeatability of the mechanical response of a single RodOri spring. **(a)** Force-displacement curves measured from three RodOri springs with identical geometry (*Lκ*_n_/2*π* = 1.32, *h*/*t* = 4, *L* = 251.3 mm, *t* = 0.6 mm). Each sample (samples 1-3) was tested three times (tests 1-3). **(b)** Enlarged view of the local unloading segments at *Δ* = 150 mm. *P*: compressive force, *Δ*: compressive displacement. *k*: spring constant.

**
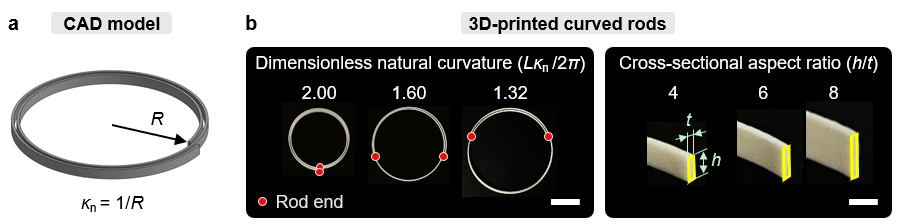
**

**Figure S2.** Design and fabrication of naturally curved elastic rods. **(a)** Spiral CAD model of a curved rod used for 3D printing. **(b)** Photographs of the 3D-printed rods with various dimensionless natural curvatures (*Lκ*_n_/2*π* = 2.00, 1.60, 1.32) and cross-sectional aspect ratios (*h*/*t* = 4, 6, 8), fabricated with fixed length (*L* = 251.3 mm) and thickness (*t* = 0.6 mm). Scale bars: 20 mm (left) and 2.5 mm (right).

**
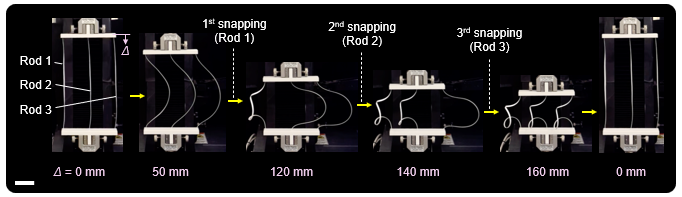
**

**Figure S3.** Sequential snapping behavior of three different types of RodOri springs under axial compression. Rod 1: *Lκ*_n_/2*π* = 2.00, *h*/*t* = 6; Rod 2: *Lκ*_n_/2*π* = 1.60, *h*/*t* = 4; Rod 3: *Lκ*_n_/2*π* = 1.32, *h*/*t* = 4. All rods are fabricated with a fixed length (*L* = 251.3 mm) and thickness (*t* = 0.6 mm). Scale bar: 5 cm.

**
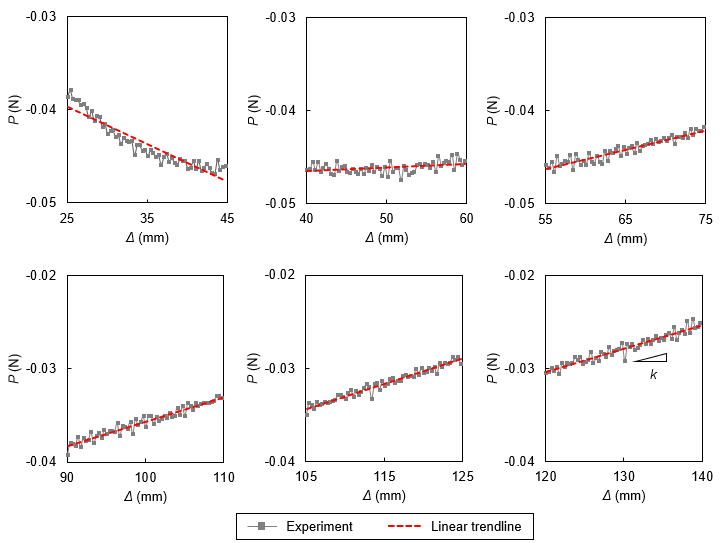
**

**Figure S4.** Local unloading responses of a single RodOri spring (*Lκ*_n_/2*π* = 1.32, *h*/*t* = 4, *L* = 251.3 mm, *t* = 0.6 mm) at different compressive displacements (*Δ* = 35, 50, 65, 100, 115, 130 mm). The spring constant *k* is extracted as the slope of each local segment via linear fitting (red dashed line).

**
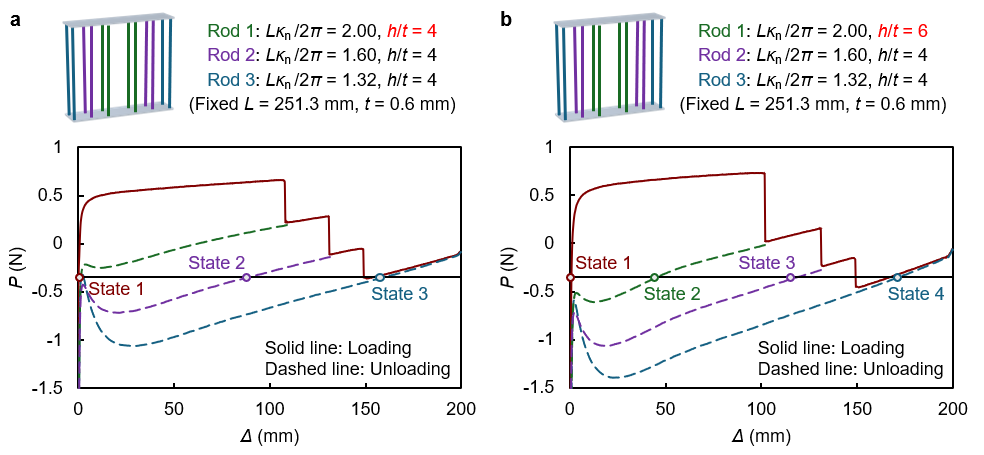
**

**Figure S5.** Force-displacement responses of RodOri spring metamaterials under axial compression with clamped-clamped boundary conditions. The mass of the rigid frame is 35 g. **(a)** Metamaterial exhibiting three stable states, composed of rods with the following parameters; rod 1: *Lκ*_n_/2*π* = 2.00; rod 2: *Lκ*_n_/2*π* = 1.60; rod 3: *Lκ*_n_/2*π* = 1.32; fixed *L* = 251.3 mm, *h*/*t* = 4, *t* = 0.6 mm. **(b)** Metamaterial exhibiting four stable states, composed of rods with the following parameters; rod 1: *Lκ*_n_/2*π* = 2.00, *h*/*t* = 6; rod 2: *Lκ*_n_/2*π* = 1.60, *h*/*t* = 4; rod 3: *Lκ*_n_/2*π* = 1.32, *h*/*t* = 4; fixed *L* = 251.3 mm, *t* = 0.6 mm.

**
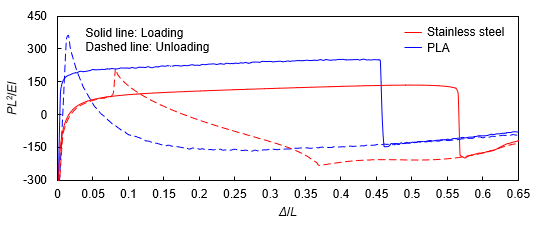
**

**Figure S6.** Normalized force-displacement curves of a single RodOri spring made of stainless steel (red) and PLA (blue). The geometric parameters are identical: *Lκ*_n_/2*π* = 2.00, *h*/*t* = 4, *L* = 628 mm, and *t* = 0.5 mm. The Young’s moduli are *E* = 2.6 GPa for PLA and *E* = 200 GPa for stainless steel. For direct comparison, compressive force (*P*) and displacement (*Δ*) are nondimensionalized as *PL*^2^/*EI* and *Δ*/*L*, respectively, where *I* = *ht*^3^/12 is the second moment of area.

**
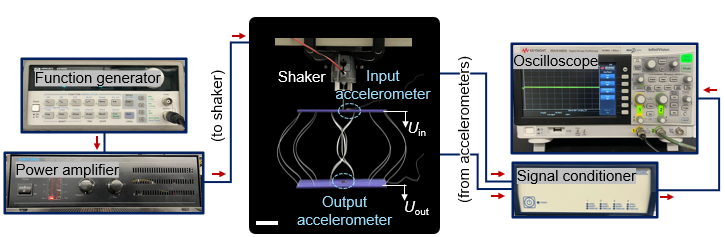
**

**Figure S7.** Schematic of the forced-vibration testing setup. The top (input) frame is excited by a modal shaker, while the bottom (output) frame remains free. Accelerometers attached to both frames measure input and output displacement amplitudes (denoted as *U*_in_ and *U*_out_). Scale bar: 5 cm.


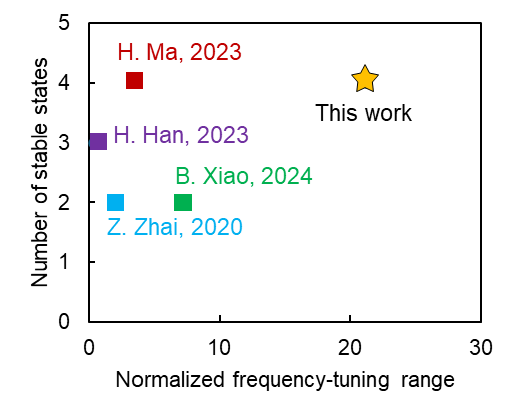


**Figure S8.** Performance comparison of the RodOri spring metamaterials with previously reported multistable metamaterials for tunable vibration control (Refs. [40-43] in the main text) in terms of the normalized frequency-tuning range and the number of stable states.

**
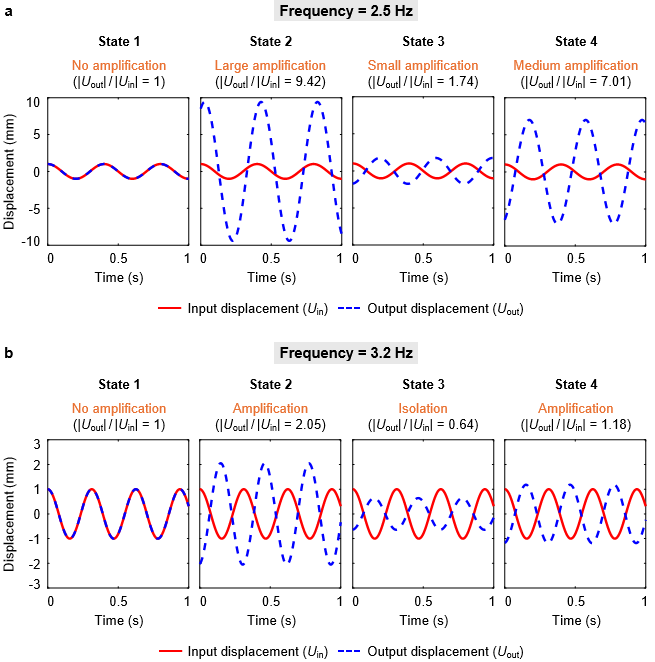
**

**Figure S9.** Numerically simulated time-domain input and output displacement signals for the four stable states of the RodOri spring metamaterial (designed in **Figure 4**) under harmonic excitation at **(a)** 2.5 Hz and **(b)** 3.2 Hz.

**
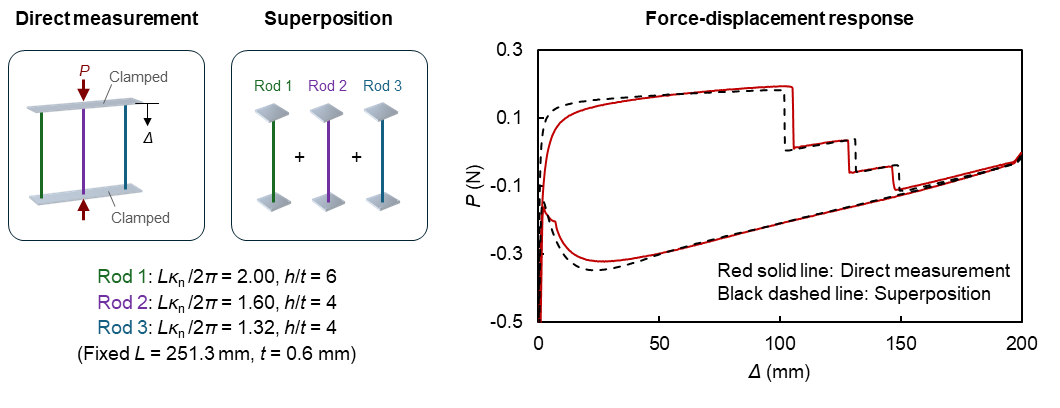
**

**Figure S10.** Comparison between the directly measured force-displacement response of a 3-rod assembly and the corresponding curve obtained by superposing the individually measured responses of the constituent rods. Rod 1: *Lκ*_n_/2*π* = 2.00, *h*/*t* = 6; rod 2: *Lκ*_n_/2*π* = 1.60, *h*/*t* = 4; rod 3: *Lκ*_n_/2*π* = 1.32, *h*/*t* = 4; fixed *L* = 251.3 mm, *t* = 0.6 mm.

**
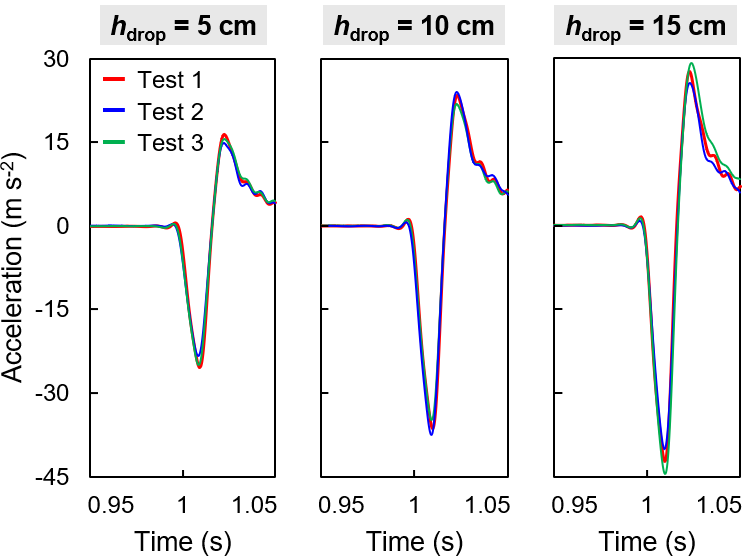
**

**Figure S11.** Repeatability of impact responses over three independent measurements. The results for the stable state 4 of the RodOri spring metamaterial (designed in **Figure 4**) are presented as an example. The acceleration signals were processed using a fourth-order low-pass Butterworth filter with a cutoff frequency of 100 Hz.

**
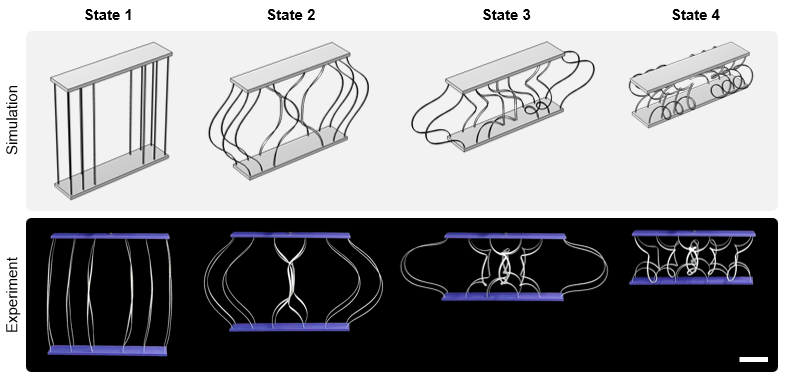
**

**Figure S12.** Configurations of the four stable states of the RodOri spring metamaterial (designed in **Figure 4**). Experimental photographs (bottom) are compared with the corresponding geometries obtained from numerical simulations (top). Scale bar: 5 cm.

**Supplementary Videos**

**Video S1.** Buckling behavior of the RodOri spring under axial compression.

**Video S2.** Reconfiguration of the bistable RodOri spring assembly between the deployed and folded states.

**Video S3.** Sequential snapping behavior of multiple RodOri springs under axial compression.

**Video S4.** Reconfiguration of the RodOri spring metamaterial among the four stable states.

**Video S5.** Demonstration of tunable vibration control using the RodOri spring metamaterial.

**References**

1. Lu, L., et al., *Multiple equilibrium states of a curved-sided hexagram: Part II—Transitions between states.* Journal of the Mechanics and Physics of Solids, 2023. **180**: p. 105407.

2. Leanza, S., J. Lee, and R.R. Zhao, *Elastic Rod Origami (RodOri) for Programming Static and Dynamic Mechanical Properties.* arXiv preprint arXiv:2510.11568, 2025.
